# Effects of* Angelica gigas* Nakai as an Anti-Inflammatory Agent in* In Vitro* and* In Vivo* Atopic Dermatitis Models

**DOI:** 10.1155/2018/2450712

**Published:** 2018-03-11

**Authors:** Seon Ok, Sa-Rang Oh, Tae-Sung Jung, Sang-Ok Jeon, Ji-wook Jung, Deok-Seon Ryu

**Affiliations:** ^1^Wellbeing Tainment Co., Ltd, Gimhae 50969, Republic of Korea; ^2^Department of Herbal Medicinal Pharmacology, College of Herbal Bio-Industry, Daegu Haany University, Kyungsan 38610, Republic of Korea; ^3^Department of Biomedical Laboratory Science, College of Medical Sciences, Soonchunhyang University, Asan 31538, Republic of Korea

## Abstract

We investigated the cellular and molecular mechanisms mediating the effects of* Angelica gigas *Nakai extract (AGNE) through the mitogen-activated protein kinases (MAPKs)/NF-*κ*B pathway using* in vitro* and* in vivo *atopic dermatitis (AD) models. We examined the effects of AGNE on the expression of proinflammatory cytokines and chemokines in human mast cell line-1 (HMC-1) cells. Compound 48/80-induced pruritus and 2,4-dinitrochlorobenzene- (DNCB-) induced AD-like skin lesion mouse models were also used to investigate the antiallergic effects of AGNE. AGNE reduced histamine secretion, production of proinflammatory cytokines including interleukin- (IL-) 1*β*, IL-4, IL-6, IL-8, and IL-10, and expression of cyclooxygenase- (COX-) 2 in HMC-1 cells. Scratching behavior and DNCB-induced AD-like skin lesions were also attenuated by AGNE administration through the reduction of serum IgE, histamine, tumor necrosis factor-*α* (TNF-*α*), IL-6 levels, and COX-2 expression in skin tissue from mouse models. Furthermore, these inhibitory effects were mediated by the blockade of the MAPKs and NF-*κ*B pathway. The findings of this study proved that AGNE improves the scratching behavior and atopy symptoms and reduces the activity of various atopy-related mediators in HMC-1 cells and mice model. These results suggest the AGNE has a therapeutic potential in anti-AD.

## 1. Introduction

Atopic dermatitis (AD) is a chronic inflammatory dermatitis disease characterized by severe itching, eczematous skin eruption, asteatosis, and red, swollen, and cracked skin [1]. The general pathogenesis of AD has not been unknown, but AD-related symptoms including repeated worsening or recurrence are primarily assumed to be caused by two reasons. One reason is the induction of allergic inflammation based on immune system disorder, called “inside-out” while the other reason is epidermal permeability barrier dysfunction induced by external stress, called “outside-in” [1–4]. Immune system disorder, especially, has been focused on as the fundamental cause of AD. An association between AD and IgE has been proven by the evidence that serum IgE increases in proportion to the symptoms of eczema, asthma, and allergic rhinitis. Moreover, serum IgE level has been shown to increase in basophils after exposure to antigens and the existence of an IgE receptor has been demonstrated in mast cell and basophils. This phenomenon is referred to as the “IgE-mediated sensitization.” However, when patients were given oral immunosuppressive drugs to improve the symptoms, they did not respond and serum IgE levels did not decrease proportionally. Factors other than IgE were subsequently identified and the condition was called “non-IgE-mediated sensitization” [5]. The original model of AD pathogenesis attributed the disease development to an imbalance in cell-mediated helper T (Th) 1 versus Th2 cell response [6]. Th1 cells primarily produce interferon- (INF-) *γ* and tumor necrosis factor-*α* (TNF-*α*), which are required for cell-mediated inflammatory reactions while Th2 cells secrete IL-4, IL-5, IL-10, and IL-13, which mediate B cell activation and IgE production in mast cells [7]. Recently, AD studies have reported that numerous mast cells are present in the skin of patients with AD [8]. Activated mast cells induced by external stress secrete vasodilatory substances including a variety of protease, histamine, and numerous cytokines, which induce protein- (e.g., TNF-*α*- and IL-6-) mediated cell signaling that subsequently induces allergic inflammation [9]. These observations provide reasonable evidence that AD precedes other allergic disease such as allergic asthma and rhinitis [10]. This suggests that, in the initial treatment of AD, it is important to cure and prevent the progression of other allergic diseases and, therefore, agents are needed to suppress mast cell activation. Recently, treatment strategies for AD including steroids, antihistaminic agents, and immunosuppressants have been developed; however, their use is limited as controlled prescription drugs because of the serious side effects associated with long-term use. Therefore, the development of natural agents as treatment options for AD with minimal side effects has been a focus.

*Angelica gigas *Nakai (AGN), a perennial umbelliferous herb, is cultivated as a medicinal plant. Depending on its geographical origin, it is called Korean, Japanese, or Chinese danggui (AGN,* Angelica acutiloba* Kitagawa, or* Angelica sinensis* Diels, resp.), which have different bioactive compounds with varying pharmacological effects. It has been mainly used to cure gynecologic diseases as well as anemia and as an immunostimulant, antioxidant, antiarthritic, and anticancer agent [11–13]. The major pharmacologically active compounds in AGN are decursin (D) and decursinol angelate (DA), which are pyranocoumarin substances only present in Korean danggui, in addition to nodakenin, nodakenetin, umbelliferon, *β*-sitosterol, *α*-pinene, and limonene [14]. Studies on the effect of AGN extracts (AGNE) have reported antihyperglycemic, neuroprotective, anti-inflammatory, and antioxidant effects and the safety of the constituents of AGNE containing 95% D/DA has been especially reported in oral acute and subacute toxicity as well as genotoxicity studies [15–18]. However, the effect of AGNE on AD has not yet been documented, despite the reported anti-inflammatory effect of D/DA.

Therefore, in this study, we investigated the effects of AGNE on the mechanisms underlying the development of AD* in vitro* and* in vivo*. Specifically, we evaluated the effects AGNE on cytokines, chemokines, and molecular signal mechanisms in AD-induced human mast cell and mice model.

## 2. Materials and Methods

### 2.1. Chemicals and Reagents

Compound 48/80, phorbol 12-myristate 13-acetate (PMA), calcium Ionophore (CI) A23187, avidin peroxidase, and DNCB were purchased from Sigma-Aldrich Chemical Corp., (St. Louis, MO, USA). IMDM was from GE Healthcare Life Science (Little Chalfont, UK) and fetal bovine serum (FBS) was obtained from JR Scientific, Inc., (Woodland, CA, USA). Anti-human IL-4/IL-10, recombinant IL-4/IL-10, biotinylated IL-4/IL-10, anti-mouse IgE, recombinant IgE, and biotinylated IgE were purchased from Pharmingen (San Diego, CA, USA). Human TNF-*α*, IL-1*β*, IL-6, IL-8, mouse TNF-*α*, and IL-6 ELISA kits were purchased from BD Biosciences (San Diego, CA, USA) and the histamine assay kit was obtained from Abnova Corp., (Taipei, Taiwan). Antibodies (Abs) were purchased from Santa Cruz Biotechnology, Inc., (Santa Cruz, CA, USA).

### 2.2. Preparation of AGNE

The AGN, which was purchased from the Ginbu GAP Farming Corp. (Pyeongchang, Gangwon, Korea) in 2017, was subsequently air-dried and stored at room temperature. Dried AGN (4 kg) was placed in nonwoven fabric and extracted with 95% ethanol at 80°C for 4 h using the machine to obtain the extract, which was then concentrated (Kyungseo, E&P, Incheon, Korea). The extract was collected, concentrated again using a rotary vacuum evaporator (KNF, Australia), and then the concentrate obtained (330 g) was designated AGNE.

### 2.3. Histamine Measurement

HMC-1 cells were seeded in 24-well plates (6 × 10^5^ cells/well) and treated with various concentrations of AGNE (0.1, 1, and 10 *μ*g/mL) for 1 h, followed by PMACI (50 *μ*M PMA + 1 *μ*g/mL CI A23187) for 4 h. The supernatants were collected, and the histamine contents secreted by the HMC-1 cells were measured using a histamine assay kit (Stressgen Biotechnologies, USA). The supernatants and histamine-alkaline phosphate conjugate (100 *μ*L each) were placed into 96-well plates for 40 min at room temperature. The plates were washed three times with PBS (0.05% Tween), and then *ρ*-nitrophenyl phosphate substrate (100 *μ*L) was added for 20 min at room temperature. The reaction was terminated by adding stop solution with 3 N sodium hydroxide (NaOH, 50 *μ*L) to the plates, followed by measurement at 405 nm using an ELISA reader (BioTek, Winooski VT, USA).

### 2.4. Isolation of Nuclear and Cytosol Fractions

The nuclear and cytosol extracts were isolated to detect NF-*κ*B p65 and I-*κ*B*α* transcription factor expression. The HMC-1 cells were seeded in a six-pi dish (6 × 10^5^ cells/dish), treated with various concentrations of AGNE (0.1, 1, and 10 *μ*g/mL) for 1 h and then PMACI (50 *μ*M PMA + 1 *μ*g/mL CI A23187) for 30 min. The dishes were washed with PBS and 400 *μ*L buffer A (10 mM 4-(2-hydroxyethyl)-1-piperazineethanesulfonic acid [HEPES] potassium hydroxide [KOH], 2 mM magnesium chloride [MgCl_2_] 0.1 mM EDTA, 10 mM potassium chloride [KCl], 1 mM DTT, and 0.5 mM phenylmethanesulfonyl fluoride [PMSF], pH 7.9). After the plates had been vortexed, they were centrifuged at 14,500 r/min. The supernatants (cytosolic fraction) were removed and the pellets (nuclear fraction) were resuspended with 300 *μ*L buffer B (50 mM HEPES/KOH, 300 mM sodium chloride [NaCl], 0.1 mM EDTA, 50 mM KCl, 1 mM DTT, 0.5 mM PMSF, and 10% glycerol, pH 7.9). After centrifuging, the supernatants (nuclear fraction) were separated.

### 2.5. Mice

Male BALB/c and ICR mice (5- and 7-week-old, resp.) were purchased from Daehan Biolink Animal Facility (Chungbuk, Korea) and housed in clean cages at Daegu Haany University during the 7 days of the experiment. Five mice were placed into each cage under controlled conditions with specific light/dark cycles twice a day. The mice were maintained at 23 ± 2°C and 55 ± 10% temperature and humidity, respectively, and were fed standard diet with water. All animal experimental protocols were approved by the Animal Ethics Committee of Daegu Haany University (DHU2016-044).

### 2.6. Compound 48/80-Induced Scratching Behavior

Compound 48/80, a reagent which locally induces itching/scratching, was used to verify the anti-itching effect of AGNE on ICR mice. AGNE (10, 20, and 40 mg/kg) and terfenadine (10 mg/kg) as a positive control were orally administered 1 h before compound 48/80 (50 *μ*g/kg) was subcutaneously injected in ICR mice. An acute toxicity test of AGNE determined that the LD50 was >2000 mg/kg. The dosage used in our experiment was determined based on the dosage verified in a previous study of the positive effects of AGNE on ulcerative colitis [19, 20]. Also, the dosage was within the range in which terfenadine does not cause adverse reactions [21]. Immediately after the itch-inducing injection, the scratching frequency was measured three times for 30 min.

### 2.7. DNCB-Induced AD

The dorsal hair of the BALB/c mice was removed, and they were left for 24 h to enable the dorsal skin to heal. Then, DNCB solutions (0.5 and 1%) were prepared by dilution with an acetone-olive oil mixture (3 : 1). To induce AD, 150 *μ*L of a 1% DNCB solution was applied to the dorsal skin for 4 days, followed by 150 *μ*L of a 0.5% DNCB solution for 3 weeks. AGNE (10, 20, and 40 mg/kg) and terfenadine (10 mg/kg) dissolved in PBS were administered orally for 2 weeks. Six symptoms (erythema, edema/papulation, oozing/crusts, excoriations, lichenification, and dryness) were assessed using a numerical scale of 0–3 based on the severity (0, no symptoms; 1, mild; 2, moderate; and 3, severe) at intervals of 1 week.

### 2.8. Cytokine Measurement

The IgE, TNF-*α*, IL-1*β*, IL-4, IL-6, IL-8, and IL-10 concentrations of the serum, skin tissues, or cell culture medium were measured using ELISA methods as described. Briefly, 96-well plates were coated with monoclonal antibodies (100 *μ*L) diluted in carbonate coating buffer (Na_2_CO_3_/NaHCO_3_) pH 9.5 for 12 h at 4°C. The plates were washed with PBS (0.05% Tween), blocked with PBS containing 1% bovine serum albumin (BSA), 5% sucrose, and 0.05% sodium azide (NaN_3_). After washing the plates, serum was added for 2 h at 37°C, the plate was cleaned, and then biotin combined with the second antibody was added for 2 h. After washing the plates, avidin peroxidase was added for 30 min at 37°C, and then the reaction solution was measured using an ELISA reader at 405 nm.

### 2.9. Histological Analysis

The mice were anesthetized with ketamine, and then the skin tissue was excised to examine the histological changes. The skin tissue samples were fixed with 10% formalin solution for 24 h, washed several times, and dehydrated, and embedded in paraffin solution for the preparation of paraffin blocks. The blocks were sliced to obtain 0.5–0.6-*μ*m sections, which were mounted on glass slides. The mounted sections were subsequently deparaffinized by treatment with xylene and stained with hematoxylin and eosin (H&E). After the washing and dehydration processes, the specimens were observed using a microscope (Olympus 71, Olympus Corp., America Inc., Center Valley, PA, USA).

### 2.10. Western Blot Analysis

HMC-1 cells were seeded into six-pi dishes (5 × 10^6^ cells/dish), treated with various concentrations of AGNE (0.1, 1, and 10 *μ*g/mL) for 1 h, followed by PMACI (50 *μ*M PMA + 1 *μ*g/mL CI A23187) for 24 h (COX-2), 2 h (pERK/ERK, pJNK/JNK, and phosphorylated-p38 (p-p38)/p38), and 30 min (NF-*κ*B p65 and I-*κ*B*α*). Following treatment, the ICR mice were euthanized, and the dorsal skin samples were isolated. The cells and tissues were lysed with radioimmunoprecipitation assay (RIPA) solution (50 mM HEPES, pH 7.4, 15 mM NaCl, 1% deoxycholate, 1 mM EDTA, 1 mM phenylmethanesulfonyl fluoride [PMSF], and 1 *μ*g/mL aprotinin). The protein concentration of the nuclear extracts, cell lysates, and tissue lysates was analyzed using the bicinchoninic acid protein assay (Pierce, Rockford, IL, USA). An equal amount of protein (20 *μ*g) of each sample was heated with 5x sample buffer (0.2 M Tris-hydrochloride [HCl], pH 6.8, 10 mM DTT, 10% SDS, 0.05% bromophenol blue, and 20% glycerol) at 100°C for 5 min. The samples were separated using 10% SDS-polyacrylamide gel electrophoresis (PAGE) and transferred onto polyvinylidene difluoride (PVDF) membrane (Millipore, MA, USA) using a semidry transfer system obtained from Bio-Rad (Hercules, CA, USA). For measuring the expression of target proteins, the PVDF membrane was blocked in 5% nonfat milk solution for 1 h and then incubated overnight at 4°C with primary antibodies against COX-2, GAPDH, p-ERK, ERK, p-JNK, JNK, p-p38, and p38. After hybridization of the primary antibody, the membrane was washed twice with 0.01 M Tris-buffered saline (pH 7.2) containing 0.1% Tween 20 (TBST) for 15 min, incubated with horseradish peroxidase-conjugated secondary antibodies (anti-rabbit and anti-goat) for 2 h, and then washed five times with TBST for 5 min. The protein expression was quantified using a western blot analysis system (Davinchi-K, Korea), followed by final detection using enhanced chemiluminescence (ECL) western blotting reagents from Santa Cruz Biotechnology Inc. (Santa Cruz, CA, USA).

### 2.11. Statistical Analysis

All the quantitative data are represented as the means ± standard deviation (SD). The significant differences were determined using an ANOVA with Student-Newman-Keuls test for the multiple comparison. A *P* < 0.05 was considered statistically significant.

## 3. Results

### 3.1. Effect of AGNE on Histamine and Cytokine Production of PMACI-Induced HMC-1 Cells

**Figure 1:**
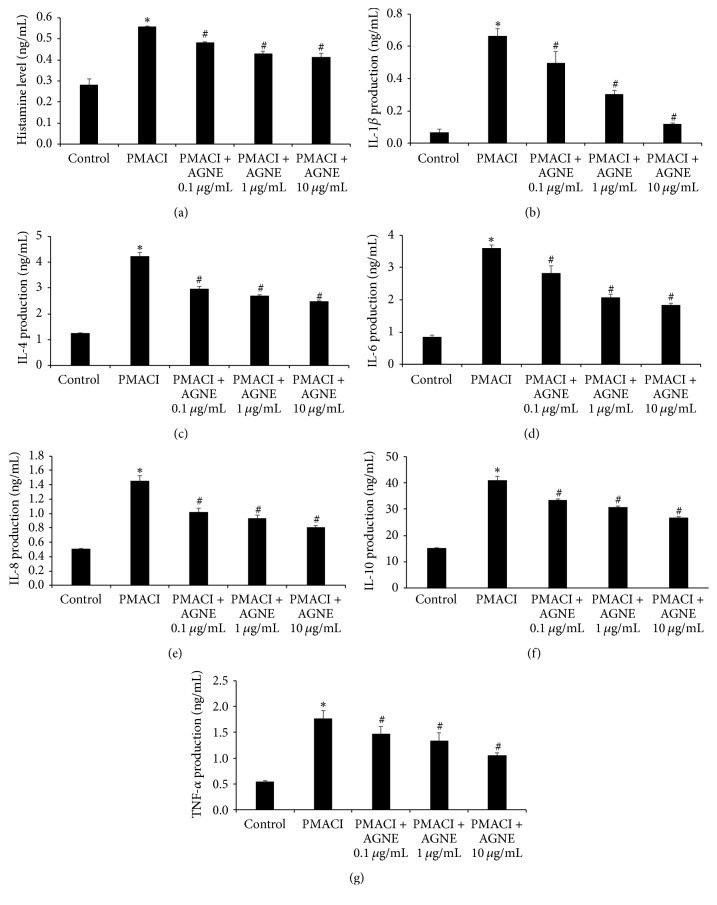
Effect of AGNE on histamine, IL-1*β*, IL-4, IL-6, IL-8, IL-10, and TNF-*α* level in PMACI-stimulated HMC-1 cells. (a) Cells (6 × 10^5^ cells/well) were pretreated with AGNE (0.1, 1, and 10 *μ*g/mL) for 1 h and then PMACI (50 *μ*M PMA + 1 *μ*g/mL CI A23187) for 4 h. Histamine content was measured using a histamine assay kit. (b–g) Cells were pretreated with AGNE for 1 h and then PMACI for 12 h. Levels of inflammatory cytokines were measured using ELISA. Data is mean ± SEM of triplicate determinations from separate triplicate experiments (^*∗*^*P* < 0.05 versus control and ^#^*P* < 0.05 versus PMACI alone).

We investigated the effect of increasing concentrations of AGNE on histamine release from PMACI-induced HMC-1 cells. Histamine release was enhanced in PMACI-stimulated cells compared to that of the control cells, but AGNE-treated cells showed a higher decrease in histamine release than PMACI-induced HMC-1 cells did. The inhibition of histamine release by AGNE was significantly dose-dependent, especially at 10 *μ*g/mL, which showed a 25.6% higher decrease than was observed in PMACI-induced HMC-1 cells (Figure 1(a)). We examined the effect of AGNE on atopy-related cytokines such as IL-1*β*, IL-4, IL-6, IL-8, IL-10, and TNF-*α*. As shown in Figures 1(b)–1(g), PMACI increased the production of cytokines (IL-1*β*, IL-4, IL-6, IL-8, IL-10, and TNF-*α*) compared to the control treatment, but AGNE pretreated cells showed a more significant dose-dependent inhibition of cytokine production than PMACI-induced HMC-1 cells did. In particular, a high concentration (10 *μ*g/mL) of AGNE showed inhibition rates of 81.9, 41.1, 49.1, 44.4, 34.6, and 39.8% for IL-1*β*, IL-4, IL-6, IL-8, IL-10, and TNF-*α*, respectively.

### 3.2. Effect of AGNE on Serum and Tissue Levels of IgE, Histamine, and Cytokine Levels on DNCB-Induced Mice Model

**Figure 2:**
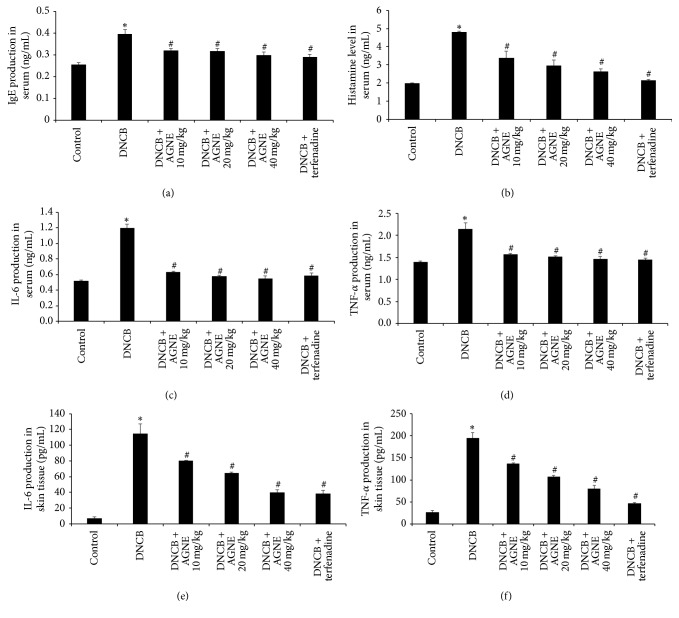
Effect of AGNE on serum and tissue levels of IgE, histamine, IL-6, and TNF-*α* in DNCB-induced mice. Blood and skin tissue samples were collected, and serum levels of (a) IgE, (b) histamine, (c) IL-6, and (d) TNF-*α* and tissue levels of (e) IL-6 and (f) TNF-*α* in indicated group were measured using ELISA and assay kit. Data is means ± SEM of triplicate determinations from separate triplicate experiments (^*∗*^*P* < 0.05 versus control group and ^#^*P* < 0.05 versus DNCB-treated group).

The IgE level is a very important index of stimulated mast cells and alleviation of the allergic response. To investigate the effect of AGNE on IgE production, a critical factor in allergy induction, we collected blood samples and analyzed the levels of serum IgE using ELISA. As shown in Figure 2(a), the results showed that serum IgE production dramatically increased in DNCB-induced BALB/c mice, but significantly decreased in the AGNE- and terfenadine-treated groups. The inhibition rates of IgE production were 18.9, 19.9, and 24.7% by AGNE 10, 20, 40 mg/kg while that by terfenadine was 26.8% compared to the DNCB-induced group. Histamine is the main cause of itching in atopy, and its release is increased in patients with AD. Therefore, we examined the effect of AGNE on histamine release by measuring its serum content in DNCB-induced BALB/c mice. As shown in Figure 2(b), the DNCB group had the highest content of histamine (4.81 ± 0.039 ng/mL) while that of the control group was 1.99 ± 0.030 ng/mL. The serum IgE contents of mice administered 10, 20, and 40 mg/kg AGNE were 3.40 ± 0.349, 2.98 ± 0.292, and 2.64 ± 0.141 ng/mL, respectively. The terfenadine group had similar histamine content (2.15 ± 0.056 ng/mL) as those of control group. The suppression rate of histamine release by AGNE 10, 20, and 40 mg/kg was 29.4, 38.2, and 45.2%, respectively, while that of terfenadine was 55.3% compared to the DNCB-induced group. AD is induced by immunological responses induced when Langerhans cells deliver allergens to lymphocytes after the IgE has recognized external allergens for the first time [10]. Cytokines are generated when dendritic cells deliver allergens to T-cells. We examined the effect of AGNE on the serum and tissue levels of representative Th1 and Th2 inflammatory cytokines such as IL-6 and TNF-*α* level increased in DNCB-induced BALB/c mice. The serum IL-6 level of mice induced with DNCB alone was 1.20 ± 0.050 ng/mL, which was decreased to 0.63 ± 0.016, 0.58 ± 0.015, 0.55 ± 0.037, and 0.59 ± 0.031 ng/mL following treatment with AGNE 10, 20, and 40 mg/kg, and terfenadine, respectively (Figure 2(c)). These levels were similar to those observed in the control group (0.52 ± 0.009 ng/mL). Moreover, the serum TNF-*α* level of 2.15 ± 0.133 ng/mL was only induced with DNCB decreased to 1.57 ± 0.020, 1.51 ± 0.027, 1.47 ± 0.052, and 1.45 ± 0.035 ng/mL following treatment with AGNE 10, 20, and 40 mg/kg, and terfenadine, respectively (Figure 2(d)). The levels of both IL-6 and TNF-*α* were significantly decreased dose-dependently by AGNE. In the AGNE 40 mg/kg group, the inhibition rate of IL-6 and TNF-*α* was 54.3 and 31.7%, respectively. To further provide the protective effect of AGNE on AD, this study measured the production level of IL-6 and TNF-*α* cytokines in the skin tissue. As shown in Figures 2(e) and 2(f), treatment with DNCB alone increased IL-6 and TNF-*α* level to 115 ± 12.033 pg/mL and 195 ± 11.746 pg/mL, respectively; on the other hand, AGNE treatment decreased dose-dependently. Especially, in the AGNE 40 mg/kg group (40 ± 3.536 pg/mL), the IL-6 level was similar to those observed in the treatment with terfenadine (38.12 ± 4.375 pg/mL). The cytokine production in the serum showed the same results in the skin tissue.

### 3.3. Effect of AGNE on Scratching Behavior, Dermatitis Sensitivity, and Histologic Changes in DNCB-Induced Mice Model

**Figure 3:**
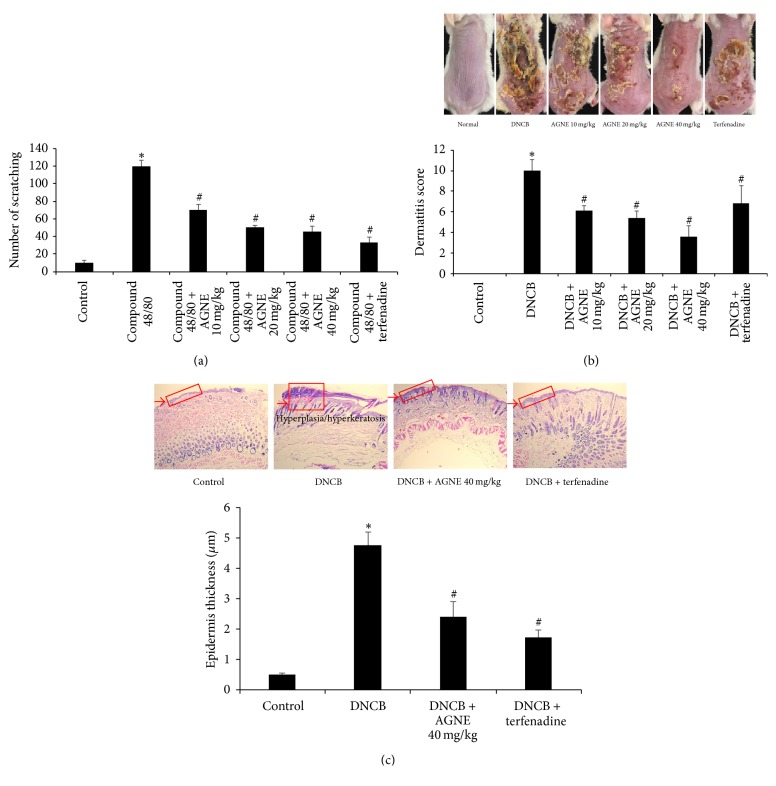
Effect of AGNE on dermatohistopathological changes in mice model. (a) AGNE (10, 20, and 40 mg/kg) was orally administered 1 h before intradermal injection of compound 48/80 (50 *μ*g/kg). Scratching behaviors were counted in ICR mice. (b) BALB/c mice were sensitized with DNCB and induced atopic dermatitis. Terfenadine was used as a positive control. (c) DNCB-induced dorsal skin was stained with H&E. Data is means ± SEM of triplicate determinations from separate triplicate experiments (^*∗*^*P* < 0.05 versus control group and ^#^*P* < 0.05 versus compound 48/80 or DNCB-treated group).

We investigated the potential inhibition of scratching behavior by AGNE in pruritus mediated by the promotion of mast cell degranulation induced by compound 48/80. AGNE (10, 20, and 40 mg/kg) and terfenadine as the positive control were orally administered 1 h before compound 48/80 (50 *μ*g/kg) was intradermally injected. The scratching behavior was counted as one incident of scratching for 30 min. The number of scratching behaviors was significantly increased by compound 48/80 compared to that of the control group, but AGNE dose-dependently and significantly blocked the number of scratches elicited by compound 48/80. In addition, terfenadine showed the strongest inhibition of scratches. The inhibition rates of total scratches in the AGNE groups were 41.2, 57.6, and 62.1%, respectively, while that of the terfenadine group was 72.5% compared with the compound 48/80-induced ICR mice (Figure 3(a)). The protective effect of AGNE on AD was investigated using an* in vivo* model induced by applying 0.5% DNCB to the dorsal skin of BALB/c twice a week for 3 weeks. Furthermore, AGNE (10, 20, and 40 mg/kg) and terfenadine were orally administered 1 week prior to the end of the experiment. We visually monitored the dorsal skin of the mice for induced atopy, and the incidence was numerically expressed using the scoring AD (SCORAD) index. The DNCB group exhibited markedly severe wounds, erosion, keratinization, and exfoliation, but the AGNE and terfenadine groups showed a reduction of these symptoms. AGNE, especially, showed a marked, dose-dependent improvement in the atopy compared with that of terfenadine (Figure 3(b)). To investigate histologic changes in AD induced by AGNE, we harvested the dorsal skin of each experimental group. The DNCB group (4.755 ± 0.432 *μ*m) exhibited marked hypertrophy of epidermal thickness, but the AGNE 40 mg/kg group (2.409 ± 0.490 *μ*m) showed an inhibition of these symptoms and improved inflammatory responses (Figure 3(c)).

### 3.4. Effect of AGNE on Inhibitory Activity of NF-*κ*B and COX-2 Expression through MAPKs Pathway in PMACI-Induced HMC-1 Cells

**Figure 4:**
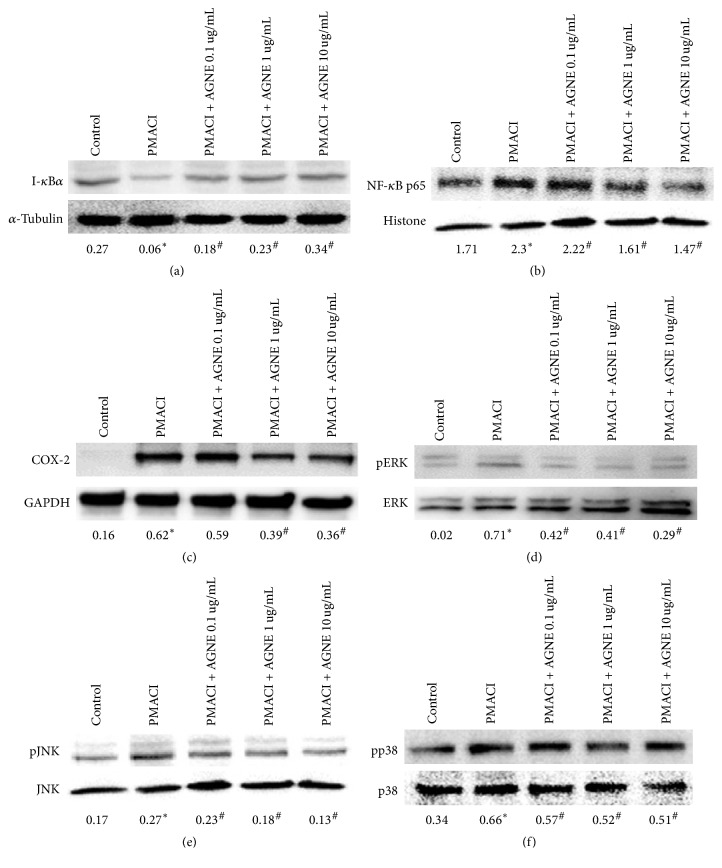
Effect of AGNE on protein expression in PMACI-induced HMC-1 cells. Cellular proteins were used to detect (a) I-*κ*B*α* phosphorylation (cytosol) and (b) DNA-binding activity of NF-*κ*B p65 (nuclear), (c) COX-2 activation, and (d) ERK, (e) JNK, and (f) p38 phosphorylation (cellular hole protein) in HMC-1 cells. Data is means ± SEM of triplicate determinations from separate triplicate experiments (^*∗*^*P* < 0.05 versus control and ^#^*P* < 0.05 versus PMACI alone or DNCB-treated group).

NF-*κ*B has been reported to be an important transcription factor in cytokine production after activation by an external stimulus. We studied I-*κ*B*α* as the negative regulator of NF-*κ*B expression, and the NF-*κ*B p65 subunit translocation to the nucleus was inhibited by AGNE as shown after isolation of the nuclear and cytosol extract. PMACI decreased I-*κ*B*α* expression in the cytoplasm due to increased expression of the NF-*κ*B*α* subunit, but AGNE significantly and dose-dependently increased I-*κ*B*α* expression. Contrary to the cytoplasm, the nuclear NF-*κ*B p65 subunit expression was increased by PMACI and significantly decreased dose-dependently by AGNE. Therefore, AGNE suppressed the PMACI-induced activation of NF-*κ*B in HMC-1 cells and, thereby, decreased cytokine release (Figures 4(a) and 4(b)). HMC-1 cells showed an increased expression of COX-2 induced by PMACI, but AGNE significantly and dose-dependently decreased the expression of COX-2 in PMACI-induced HMC-1 cells (Figure 4(c)). We examined the phosphorylation of ERK, JNK, and p38 of HMC-1 cells to determine the effect of AGNE on the MAPK signaling pathway. PMACI significantly induced the levels of p-ERK, p-JNK, and p-p38 versus ERK, JNK, and p38 in HMC-1 cells while AGNE dose-dependently reduced the levels of these phosphorylated molecules stimulated by PMACI (Figures 4(d)–4(f)).

### 3.5. Effect of AGNE on COX-2 Expression through MAPKs Pathway in DNCB-Induced Mice Model

**Figure 5:**
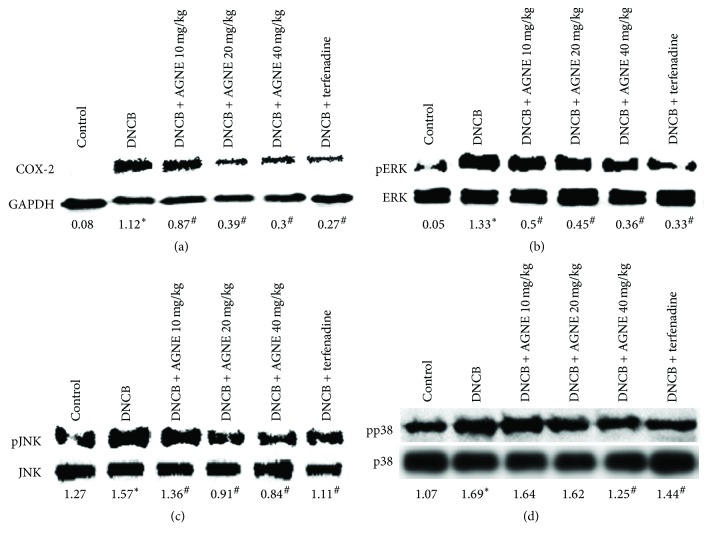
Effect of AGNE on protein expression in DNCB-induced BALB/c mice. Total protein tissue samples were collected to detect (a) COX-2 activation and (b) ERK, (c) JNK, and (d) p38 phosphorylation in mice model. Data is means ± SEM of triplicate determinations from separate triplicate experiments (^*∗*^*P* < 0.05 versus control and ^#^*P* < 0.05 versus PMACI alone or DNCB-treated group).

Inhibition of DNCB-activated COX-2 expression by AGNE in mice was examined using western blot analysis. As shown in Figure 5(a), the control group expressed no detectable levels of COX-2 protein, but COX-2 expression was markedly increased in response to DNCB, while treatment with AGNE (10, 20, or 40 mg/kg) suppressed the DNCB-induced COX-2 expression levels in a dose-dependent manner. The inhibition rate of COX-2 expression was 22.5%, 64.5%, 73.5%, and 76.3% by AGNE 10, 20, and 40 mg/kg and terfenadine, respectively. The effects of AGNE on the phosphorylation of MAPKs such as ERK, JNK, and p38 were analyzed to elucidate the underlying mechanisms of the inflammatory response. The MAPKs signaling pathways play a critical role in the regulation of inflammatory responses and coordinating the induction of numerous gene-encoding inflammatory mediators. Phosphorylation of ERK, JNK, and p38 increased in the DNCB-induced mouse model, whereas AGNE dose-dependently and significantly suppressed the phosphorylation of ERK, JNK, and p38. The inhibition rate of the 40 mg/kg AGNE group was 73.4%, 46.2%, and 25.6% for p-ERK, p-JNK, and p-p38, respectively, compared with the DNCB-induced group. Terfenadine, an antihistamine medicine, which was used as the positive control, suppressed phosphorylation of ERK, JNK, and p-38 by 75.5%, 29.5%, and 14.7%, respectively, similar to 40 mg/kg AGNE (Figures 5(b)–5(d)).

## 4. Discussion

The bioactive compounds in AGN are D, DA (a constitutional isomer of D), decursinol, and nodakenin similar to coumarin, especially D, DA, and nodakenin, which are known to have anti-inflammatory effects. In addition to the complex medicinal herbs included in AGN, Gamisasangja-tang or decursin derivatives suppress pruritus and atopic skin inflammation in AD mouse models [22–27]. However, the effect and underlying molecular mechanism of the D/DA-rich AGNE have not been evaluated in AD* in vitro* and* in vivo*. In this study, we analyzed the components of AGNE and discovered it contains 50% D/DA, 48% polysaccharide, and 2% nodakenin [20]. The particular constituent mediating the effect of this extract on AD has not been clarified, but we speculate that it is likely the coumarin compounds, which affected AD in HMC-1 cells and the DNCB-induced BALB/c mice model. We will need to further study the isolated coumarin derivatives such as D, DA, and nodakenin from AGN and their effects on AD models. Terfenadine, which was used as a positive control, is an antihistamine previously used for the treatment of allergic conditions.

AD is a chronic, allergic inflammatory skin disease characterized by eczema and scratching behavior, which can induce skin injury. PMACI has been evaluated as allergen related to the production of various cytokines (IL-1*β*, TNF-*α*, IL-6, and IL-8) and release of IgE and histamine in mast cells. Compound 48/80 is widely used in animal and tissue models as a “selective” mast cell activator and can induce scratching behavior in mice [28]. In addition, DNCB, which is a contact dermatitis allergen similar to 2,4-dinitrofluorobenzene (DNFB) and ovalbumin, induces an allergic immune response of AD lesions on mouse skin. The allergens, PMACI, compound 48/80, and DNCB were used to establish AD models* in vitro* and* in vivo* and specifically induced each biomarker related to the AD model from 2- to 10-fold compared to the control. These results are a similar fold to that of the control of other studies using the same models [29–31]. DNCB-induced mice, especially, showed deep external wounds, erosion, cornification, and exfoliation of dead skin cells, but the AGNE-treated mice exhibited an obvious alleviation of symptoms. The skin histological analysis revealed hyperkeratosis on the skin surface of DNCB-induced mice; however, this was relieved by AGNE treatment. Scratching behavior occurs in the affected area due to pruritus induced in the broken skin barrier and the affected area is aggravated by repeated itching, because nonideally activated Th2 cells, as well as immune responses of mast cells, are involved [32]. AGNE significantly suppressed the number of scratching behaviors compared to that observed in the compound 48/80-induced mice. Skin inflammation is characterized by increased serum IgE levels, expression of Th1/Th2 cytokine, or accumulation of inflammatory cells. According to various studies, serum IgE levels are elevated in most patients with AD, and IgE levels increase as the severity of AD increases [33]. It was recently suggested that IgE levels may potentially be used to predict the prognosis of AD, and they have been used as supplementary information for the diagnosis of AD [34]. AGNE treatment significantly decreased the serum IgE level of DNCB-induced mice. The initial response in various skin diseases involving atopy is mediated by histamine release in mast cell and; therefore, antihistamine drugs are commonly used for treating AD. Histamine is a well-known compound secreted by degranulation of mast cell and is an important factor involved in the immediate hypersensitivity reaction. Mast cells have a key role in biophylaxis associated with innate and acquired immunity; however excessive activation of mast cells increases inflammation as well as secretion of histamine and serotonin, which trigger itching in addition to the production of proinflammatory cytokines such as TNF-*α* and IL-6 [9]. Mast cells activated by PMACI, especially, produce Th2 cytokines such as IL-4 and react to IgE as the causative cell [35, 36].

In this study, we examined the potential modulatory effect of AGNE on histamine release in activated HMC-1 cells and DNCB-induced mice. The results demonstrated that AGNE relieved itching and heat by inhibiting histamine release. In addition, we investigated the inhibitory effect of AGNE on inflammatory cytokines (TNF-*α*, IL-1*β*, IL-6, and IL-8) in PMACI-induced HMC-1 cells and TNF-*α* and IL-6 in DNCB-induced mice. Increased IL-4 synthesis is induced by IgE in B cell after overproduction of IL-4 and IL-10 by functional reduction such as inhibition of Th2 cell proliferation when some antigen exposure occurs in the blood of patients [37]. AGNE dose-dependently inhibited the production of cytokines (IL-4 and IL-10) in PMACI-induced HMC-1 cells. These results suggest that AGNE directly suppressed the activation of Th2 cells. IL-1*β*, a cytokine that induces inflammation, is produced by macrophages, neutrophilic, leukocytes, epithelial cells, and endothelial cells and is increased by other cytokines such as TNF-*α* or bacterial lipopolysaccharides [38]. IL-6, an inflammatory cytokine secreted by immune cells such as T-cells, monocytes, macrophages, and synovial fibroblasts, is increased in humans following stimulation by inflammation, infection, and external injuries [39]. IL-8, a chemokine known as a neutrophil chemotactic factor, induces chemotaxis in target cells and migrates to the site of infection in humans.

Taken together, the deterioration of skin lesions observed in DNCB-induced mice model is due to increased IgE production in serum and skin tissue, as well as degranulation of mast cells caused by an increased mast cell population [40]. A previous study reported that* Angelica gigas* Nakai extract inhibits Th2 cell differentiation [41]. TNF-*α* controls epidermal growth and induces the activation of the signaling molecule NF-kB to stimulate Th2 cytokines such as IL-4 and IL-6 and subsequently increase IgE switching. This increases the level of IgE and the subsequent symptoms of AD, such as hyperplasia and acanthosis in AD mice model. It is also known to accelerate mast cell degranulation [42].

COX-2, a protein related to inflammation and immune function, is involved in the arachidonic acid cascade and is converted to prostaglandin and leukotriene [43]. In AD, the ceramide barrier of the skin is broken [44]. Ceramide derivatives produce inflammatory mediators through the arachidonic cascade after the skin barrier is damaged by an imbalance of the Th1/Th2 immune system and ceramide derivatives. AGNE dose-dependently reduced the expression of COX-2 in PMACI-induced HMC-1 cells and DNCB-induced mice. Inflammation is initiated following external irritation-induced expression of transcription factors such as activator protein-1 (AP-1) or NF-*κ*B by phosphorylation of MAPKs [35, 45]. NF-*κ*B is considered a critical transcription factor that is regulated by initial inflammatory reactions due to the expression of genes such as chemokines and adhesion molecules for transporting immune cells or inflammatory cytokines such as TNF-*α*, IL-1*β*, IL-6, and IL-8. I*κ*-B*α* binds to and inhibits NF-*κ*B by sequestering it in an inactive state in the cytoplasm, and NF-*κ*B subsequently translocates to the nucleus when I*κ*-B*α* is isolated from NF-*κ*B. In this study, we demonstrated that AGNE effectively blocked the migration of NF-*κ*B to the nucleus by isolating I*κ*-B*α* in the cytosol. These results suggest that AGNE suppressed inflammatory responses in AD by reducing the secretion of inflammatory cytokines such as TNF-*α*, IL-1*β*, IL-6, and IL-8 blocking the NF-*κ*B pathway. MAPKs play an important role in the induction of inflammatory mediator production after activation by external infection. JNK and p38 kinase induce biological reaction such as apoptosis, cell differentiation, and inflammation mediated by inflammatory cytokines such as IL-1*β* and TNF-*α* ERK is involved in signal transduction, which promotes the differentiation or growth of cell. In this study, as the production of cytokines decreased, phosphorylation of MAPKs decreased following AGNE treatment.

In conclusion, our findings showed that AGNE reduces the production of histamine and atopy-related cytokines in HMC-1 cells and histamine, serum IgE, and proinflammatory cytokines in mice model. Also, our data suggest that AGNE mitigates the scratching behavior and atopy symptoms and inhibits the histologic changes. These effects are mediated by the suppression of COX-2, NF-*κ*B, and I*κ*-B*α* levels through MAPK signaling pathways. Therefore, AGNE may be an ideal oral anti-AD agent for treatment.
